# In Vitro Evaluation of ALDH1A3-Affinic Compounds on Breast and Prostate Cancer Cell Lines as Single Treatments and in Combination with Doxorubicin

**DOI:** 10.3390/cimb45030139

**Published:** 2023-03-06

**Authors:** Osama H. Abusara, Ali I. M. Ibrahim, Hamzah Issa, Alaa M. Hammad, Worood H. Ismail

**Affiliations:** 1 Faculty of Pharmacy, Al-Zaytoonah University of Jordan, Amman 11733, Jordan; 2 Aurum Biotech, Amman 11941, Jordan

**Keywords:** ALDH inhibitors, doxorubicin, combination treatment, breast cancer, prostate cancer

## Abstract

Aldehyde dehydrogenase (ALDH) enzymes are involved in the growth and development of several tissues, including cancer cells. It has been reported that targeting the ALDH family, including the ALDH1A subfamily, enhances cancer treatment outcomes. Therefore, we aimed to investigate the cytotoxicity of ALDH1A3-affinic compounds that have been recently discovered by our group, on breast (MCF7 and MDA-MB-231) and prostate (PC-3) cancer cell lines. These compounds were investigated on the selected cell lines as single treatments and in combination with doxorubicin (DOX). Results showed that the combination treatment experiments of the selective ALDH1A3 inhibitors (compounds **15** and **16**) at variable concentrations with DOX resulted in significant increases in the cytotoxic effect on the MCF7 cell line for compound **15**, and to a lesser extent for compound **16** on the PC-3 cell line, compared to DOX alone. The activity of compounds **15** and **16** as single treatments on all cell lines was found to be non-cytotoxic. Therefore, our findings showed that the investigated compounds have a promising potential to target cancer cells, possibly via an ALDH-related pathway, and sensitize them to DOX treatment.

## 1. Introduction

Aldehyde dehydrogenases (ALDHs) are phase I oxidizing enzymes [1], which irreversibly oxidize endogenous or exogenous short-chain aliphatic or aromatic aldehydes to their corresponding carboxylic acids [2,3]. Human ALDHs are NAD(P)+-dependent enzymes that occur in 19 different isoforms [1,4,5]. ALDH oxidation reactions may result in either detoxification of aldehydes or production of bioactive products, such as retinoic acid (RA), which is involved in signaling pathways that are important for the growth and development of many organs and tissues [3,6].

Furthermore, the expression of ALDHs is linked to the cancer progression of several tissues, such as melanoma and melanoma stem cells, lung cancers, human prostate cancer, bone metastasis, hepatocellular carcinoma and adenoma, and breast cancer [7,8,9,10,11,12]. The ALDH isoforms include the ALDH1A subfamily (ALDH1A1, ALDH1A3), ALDH3A1, ALDH7A1, and ALDH18A1. In addition, ALDH-active cells have been identified as biomarkers for tumor-initiating and metastasis-initiating cells in human prostate cancer [13]. The ALDH2 subfamily is also associated with alcohol-related cancers [14] and its polymorphisms increase the risk of esophageal cancers [15].

Moreover, ALDHs are expressed in normal stem cells (NSCs) and cancer stem cells (CSCs) and, hence, have been considered as CSC biomarkers [16,17]. ALDH1A1, ALDH1A3, and ALDH3A1 are among the ALDH isoforms that are important in self-protection, differentiation, and cellular proliferation of NSCs and CSCs [18,19]. Thus, targeting ALDH enzymes has a high potential for cancer treatment and diagnosis [20].

**Figure 1:**
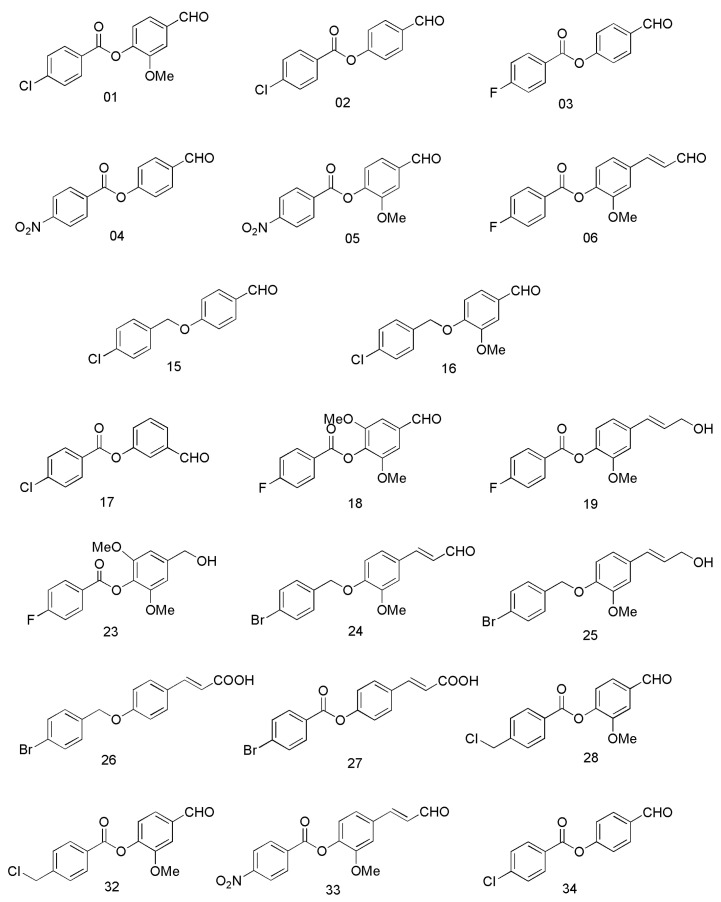
Chemical structures of ALDH-affinic compounds, adapted from Ibrahim et al. [21].

Recently, our group has discovered a number of compounds with ALDH-affinic properties (Figure 1), compounds **1**–**6**, **15**–**19**, **23**–**28**, and **32**–**34**, which have been investigated on several ALDH-expressing cancer cell lines and have shown promising cytotoxic results [21,22]. These compounds have been found to work via a variety of mechanisms, ranging from inhibiting to activating particular ALDH isoforms [21,22]. In particular, compound **15** was the most potent ALDH1A3 inhibitor, followed by compounds **16**, **18**, and **1,** with remaining enzyme activities of 0.14%, 4.27%, 16.01%, and 21.07%, respectively, compared to the control enzyme activity [21]. In these studies, non-small cell lung cancer cell lines (NSCLC) (A549 and H1299) [21] and prostate cancer cell lines (PC-3, LNCaP, and DU145) [22] have been used. A549 has been shown to express ALDH1A1, ALDH1A3, and ALDH3A1 isoforms [23,24], which were found to be absent in H1299 [24]. PC-3 has been shown to express ALDH1A1, ALDH1A3 [25], and ALDH3A1 [22]; LNCaP has been shown to express the ALDH1A3 isoform [22,25], and DU145 has been shown to express ALDH1A1 and ALDH1A3 isoforms [22].

Further investigation is required to demonstrate the potential of these ALDH-affinic compounds in cancer treatment. This includes exposing additional cancer cell lines to these compounds, either as single agents or in combination with anticancer drugs to enhance their sensitivity.

Although doxorubicin (DOX) is a commonly prescribed anticancer drug for a wide variety of cancer types, emerging resistance is considered a major barrier to its effective treatment outcomes. Several studies have shown that high ALDH activity and/or expression is correlated with low treatment outcomes using DOX [7,26,27,28]. In addition, lower expression, or downregulation of ALDH, has been found to enhance the treatment consequences for DOX [7]. Therefore, combining the ALDH-affinic compounds with DOX and measuring the consequent effects on several cancer cell lines is a promising line of investigation.

Breast cancer cell lines have been reported to express ALDH1A3 with a variety of in vitro activities, with MDA-MB-468 having the highest ALDH activity, followed by SKBR3, MDA-MB-435, BT-20, MCF7, T47D, and finally MDA-MB-231 [12]. This activity was measured via an Aldefluor assay, showing that MCF7, T47D, and MDA-MB-231 cell lines are among the breast cancer cells with low Aldefluor activity, while SKBR3 is among those with high Aldefluor activity [12]. Similar results have been observed by Charafe-Jauffret et al. [29], who reported that T47D, MCF7, and MDA-MB-231 cell lines have 0–1% of the tumorigenic Aldefluor+ cells, whereas the SKBR3 cell line has 100% of the Aldefluor+ cells. The ALDH1A1 isoform has also been shown to promote angiogenesis via vascular endothelial growth factor in vitro and in vivo using the MCF7 cell line [30]. Hence, there is the possibility that ALDH1A1 is involved in breast cancer progression and diffusion [30]. Additionally, the ALDH3A1 isoform is expressed in the MCF7 cell line, but with low activity. This isoform can be overexpressed using various drug treatments, as determined by mRNA levels [31].

In addition, using diethylaminobenzaldehyde (DEAB), which is a pan-ALDH inhibitor [32], has been found to sensitize MDA-MB-468 and MDA-MB-231 cells to DOX, paclitaxel, and radiation, with a significant reduction in cell viability compared to DOX, paclitaxel, or radiation alone [33]. Moreover, combination therapy with DOX is used in the treatment of breast cancer to achieve a synergistic effect [34].

In the present study, we aimed to investigate the cytotoxicity of DEAB and the recently discovered ALDH-affinic compounds [21] on several ALDH1A3-expressing breast and prostate cancer cell lines as single agents, and in combination with DOX to evaluate the possibility of enhancing sensitization to DOX treatment.

## 2. Results and Discussion

### 2.1. The Expression of ALDH1A3 in MCF7, MDA-MB-231, and PC-3 Cell Lines

**Figure 2:**
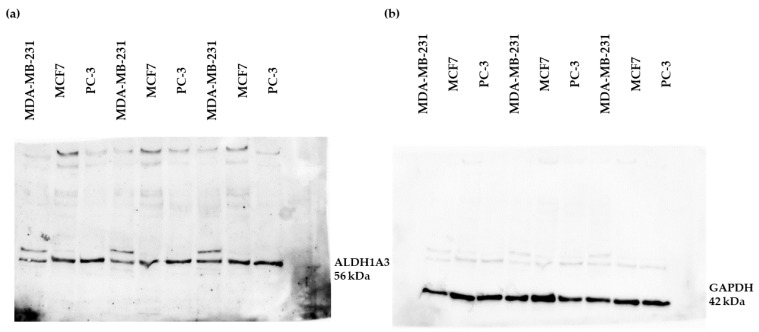
Western blot membranes showing the expression of (**a**) ALDH1A3 enzyme and (**b**) GAPDH loading control on MDA-MB-231, MCF7, and PC-3 cell lines.

As discussed in the introduction, breast cancer (MCF7 and MDA-MB-231) and prostate cancer (PC-3) cell lines are known to express ALDH1A3. In our study, we also investigated the expression of this enzyme in these cell lines before conducting our experiments. As shown in Figure 2, these cell lines were confirmed to express the ALDH1A3 enzyme and were, therefore, considered suitable for use in the cell viability experiments. As the figure shows, there might be a variation in the expression of ALDH1A3 enzyme in these cell lines, in line with previous work reported in the literature [12,22,29].

### 2.2. Cell Viability Assays

Cell viability was evaluated using an MTT colorimetric assay by treating MCF7, MDA-MB-231, and PC-3 cell lines for 48 h with compounds **1**–**6**, **15**–**19**, **23**–**28**, and **32**–**34**, which have been synthesized by our group [21], and DEAB as a positive control. The results are shown in Table 1.

The IC_50_ values presented in Table 1 showed that compound **24** is the most cytotoxic on all cell lines tested: MCF7, MDA-MB-231, and PC-3, with IC_50_ values of 24.5, 31.7, and 25.2 μM, respectively. In contrast, compounds **1**–**5**, **15**–**18**, **23**, **34**, and DEAB showed no cytotoxicity in any of the cell lines tested. Compounds **19** and **27** showed no cytotoxicity in MDA-MB-231 and PC-3 cell lines, while compound **33** was non-toxic in the MCF7 cell line, with IC_50_ values higher than 100 μM. The IC_50_s of the remaining compounds on the indicated cell lines are shown in Table 1; these are all higher than the values obtained for compound **24**.

Ibrahim et al. [21] have reported that compounds **15** and **16** were the most potent of these ALDH1A3 inhibitors, with IC_50_ values of 0.23 μM and 1.29 μM, respectively. Given that compounds **15** and **16** showed no cytotoxicity on the tested cell lines, which have been found to variably express the ALDH1A3 isoform—as previously mentioned and as our Western blot results showed (Figure 2)—a possible explanation is that these compounds were detoxified by other isoforms or other oxidoreductases [21]. Hence, compounds **15** and **16** may act as substrates for other ALDH isoforms, thus affecting their antiproliferative activity [21].

Compounds **2**, **5**, **6**, **17**, and **19** have not shown significant inhibition of ALDH1A3 activity, in contrast to compounds **1** and **18** [21]. However, compound **6** was found cytotoxic on all tested cell lines (MCF7, MDA-MB-231, and PC-3) with IC_50_ values of 47.0, 57.9, and 50.7 μM, respectively, as presented in Table 1. Compound **19** showed an IC_50_ value of 91.9 μM on the MCF7 cell line, with no cytotoxicity in either MBA-MB-231 or PC-3 cell lines (Table 1). The mechanism of how these compounds affect cell proliferation warrants further investigation.

Compounds **24**–**26**, **28**, and **32** showed cytotoxic effects on both MCF7 and PC-3 cell lines, compound **27** was cytotoxic in MCF7 cells only, and compound **33** was cytotoxic in MDA-MB-231 and PC-3 cell lines, as presented in Table 1. It is worth mentioning that according to Ibrahim et al., compounds **24**, **28**, **32**, and **33** have shown variable cytotoxicity on A549 and H1299 cell lines [21]. Again, these cytotoxic effects are worth further investigation.

Compounds **6**, **24**, and **33** are derivatives of coniferyl aldehyde that contain cinnamaldehyde nuclei [21]. Cinnamaldehyde has been reported to be cytotoxic to cancer cell lines, such as leukemia K562 [35] and MCF7 [36]. Although previous studies have reported that coniferyl aldehyde derivatives are non-toxic on MCF7 and NCI-H187 (small-cell lung cancer) cell lines [37], compounds **6**, **24** and **33** were found to be cytotoxic against MCF7 (compounds **6** and **24** only), MDA-MB-231, and PC-3 cell lines, as presented in Table 1.

Ibrahim et al. [21] reported that several ALDH-affinic compounds (**6**, **17**, **19**, **24**, **28**, **32**, and **33**) resulted in cytotoxicity in H1299 cells, which do not express the ALDH1A3 isoform. On the other hand, on A549 cells—an ALDH1A3-expressing cell line—a cytotoxic effect was reported only for compounds **23**, **28**, and **32** [21]. The lack of cytotoxicity of ALDH1A3 potent inhibitors in this study and the finding from our previous study [21] that there was no cytotoxicity on ALDH1A3-expressing cells, shows that the cytotoxicity mechanism is not necessarily related to ALDH1A3 inhibition. Other mechanisms might be involved in the cytotoxicity of these ALDH-affinic compounds, which requires further investigation.

### 2.3. Combination Treatments with Doxorubicin

Variable sensitivity to DOX has been reported in breast and prostate cancer cell lines [38,39,40]. In addition, the expression of ALDHs is related to decreased cytotoxic effect and sensitivity to DOX [7,26,27,28], as mentioned above.

To investigate enhancing DOX sensitivity via an ALDH-related pathway, selected compounds were tested in this study in combination with DOX on MCF7 and PC-3 cell lines. These cell lines were chosen because they express ALDH1A3 isoform. Compounds **15**, **16**, **24**, **27**, **33**, and DEAB were used in this study, in which compounds **15** and **16** have been found to be the most potent ALDH1A3 inhibitors [21] and, according to the IC_50_ values shown in Table 1, they were considered relatively non-cytotoxic. Compound **24** showed the highest cytotoxicity on all tested cell lines, while compounds **27** and **33** appeared to be non-cytotoxic on PC-3 and MCF7 cell lines (Table 1), respectively. DEAB, a known pan-ALDH inhibitor [32], was chosen to evaluate any enhancement of DOX sensitivity in combination treatments.

Cell viability assays were also evaluated via MTT colorimetric assays by treating MCF7 and PC-3 cell lines for 48 h with compounds **15**, **16**, **24**, **27** (on PC-3), and **33** (on MCF7), in combination with DOX. In addition, DEAB was used in combination with DOX on MCF7 and PC-3 cell lines. The concentrations used for DOX were selected based on their respective IC_50_ on each cell line: 5 μM and 1 μM for MCF7 and PC-3, respectively.

For compounds **15** and **16**, concentrations of 90, 60, 30, 10, and 6 μM were used, which were below their IC_50_ values on both MCF7 and PC-3 cell lines. This was to investigate whether the combination treatment showed a dose-dependent sensitization or whether the effect was independent of the concentration used. For compound **24**, the concentrations used (100, 50, 25, 12.5, and 6.25 μM) ranged above and below its respective IC_50_ value (~25 μM, Table 1) on MCF7 and PC-3 cell lines. This was in order to evaluate the effect of cytotoxic and non-cytotoxic concentrations on cell viability when used alone or in combination with DOX. For compounds **27** and **33**, several concentrations were used (160, 80, 40, 20, and 10 μM). As compounds **27** and **33** have expected IC_50_ values above 100 μM, 160 μM was used alongside concentrations below 100 μM to determine the effect of its non-cytotoxic concentrations, as with compounds **15** and **16**. Similar to compounds **27** and **33**, the concentrations used for DEAB were 100, 50, 25, 12.5, and 6.25 μM, which are non-cytotoxic concentrations on both cell lines.

**Figure 3:**
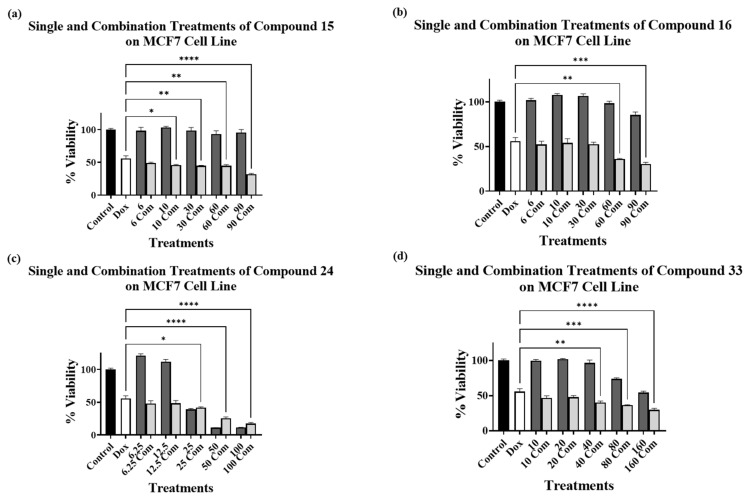
Cell viability assays using compounds (**a**) **15**, (**b**) **16**, (**c**) **24**, and (**d**) **33** alone at various concentrations (μM) (dark grey bars) and in combination with 5 μM DOX (light grey bars) on MCF7 cell line. Control bar chart (black bar) means no treatments were added. DOX at 5 μM alone is presented as a white bar. Experiments were performed in triplicates at three independent experiments with controls (* *p* < 0.05, ** *p* < 0.01, *** *p* < 0.001, and **** *p* < 0.0001).

**Figure 4:**
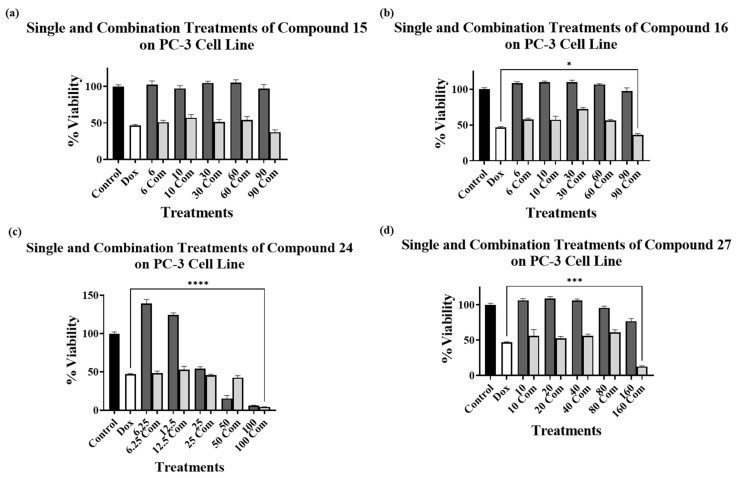
Cell viability assays using compounds (**a**) **15**, (**b**) **16**, (**c**) **24**, and (**d**) **27** alone at various concentrations (dark grey bars) and in combination with 1 μM DOX (light grey bars) on PC-3 cell line. Control bar chart (black bar) means no treatments were added. DOX at 1 μM alone is presented as a white bar. Experiments were performed in triplicates in three independent experiments with controls (* *p* < 0.05, *** *p* < 0.001, and **** *p* < 0.0001).

**Figure 5:**
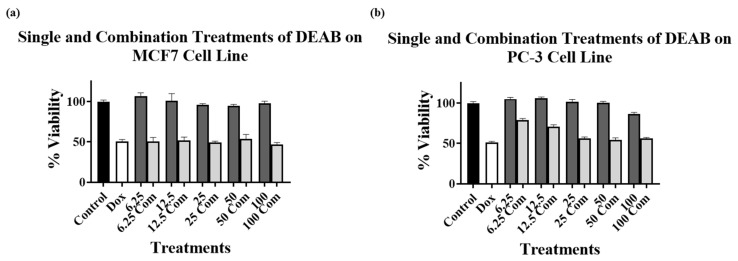
Cell viability assays using DEAB on (**a**) MCF7 and (**b**) PC-3 cell lines alone at various concentrations (dark grey bars) and in combination with (**a**) 5 μM DOX and (**b**) 1 μM DOX (light grey bars). Control bar charts (black bars) mean no treatments were added. DOX at (**a**) 5 μM and (**b**) 1 μM alone are presented as white bars. Experiments were performed in triplicates in three independent experiments with controls.

One-way ANOVA followed by Tukey’s multiple comparisons analysis was conducted to analyze the results of the combination treatments on MCF7 and PC-3 cell lines when compared to DOX alone to determine any significant enhancement of cytotoxicity, as shown in Figure 3, Figure 4 and Figure 5.

Figure 3a–d show the cytotoxic effect of compounds **15**, **16**, **24** and **33**, respectively, in combination with 5 μM DOX compared to 5 μM DOX treatment alone on the MCF7 cell line. The data analysis revealed a significant decrease in cell viability of the cells treated with 5 μM DOX in combination with various concentrations of compound **15** (Figure 3a), with 60 and 90 μM of compound **16** (Figure 3b), with 25, 50, and 100 μM of compound **24** (Figure 3c), and with 40, 80, and 160 μM of compound **33** (Figure 3d). The combination of compound **15** with DOX showed a significant reduction in MCF7 cell viability (Figure 3a) at all concentrations except for 6 μM. The significant reduction of cell viability for compound **16** was only observed at higher concentrations (60 μM and 90 μM), compared to compound **15**, as shown in Figure 3b.

Taking into consideration that compounds **15** and **16** were found to be non-cytotoxic on MCF7 cells (Table 1) and were the most potent ALDH1A3 inhibitors, with compound **15** being more potent than **16** [21], it could be hypothesized that compound **15** sensitized MCF7 cells to DOX treatment to a greater extent than compound **16**. Voulgaridou et al. have reported that MCF7 cells expressing the ALDH3A1 isoform resulted in approximately 11-fold resistance to DOX compared to ALDH3A1 non-expressing cells [41]. Therefore, a possible explanation for this enhancement of DOX cytotoxicity could be that compounds **15** and **16** target the ALDH enzymes as either inhibitors (for ALDH1A3) or potential substrates (for ALDH3A1) [21], which could lead to sensitization of the MCF7 cells to DOX treatment. Compounds **24** and **33** also showed significant reduction in cell viability compared to DOX alone (Figure 3c,d), but only at relatively high concentrations, which may indicate a non-sensitizing combined cytotoxic activity.

Figure 4a–d show the cytotoxic effect of compounds **15**, **16**, **24**, and **27**, respectively, on PC-3 cells, in combination with 1 μM DOX compared to the single 1 μM DOX treatment. The results showed no decrease in the cell viability for the combination treatment of DOX with compound **15** at any of the concentrations used (Figure 4a). However, a significant decrease in cell viability for the combination treatments with DOX was observed with compound **16** at 90 μM (Figure 4b), with compound **24** at 100 μM (Figure 4c), and with compound **27** at 160 μM (Figure 4d).

The results obtained from the combination treatment experiments on the PC-3 cell line showed a significant reduction in cell viability only at the highest concentration used for the compounds tested (**16**, **24**, and **27**) in combination with DOX, compared to DOX alone. Unexpectedly, compound **15**, at all tested concentrations, did not show any enhancement of the DOX cytotoxicity, compared to the single DOX treatment. A possible explanation for the differences observed between MCF7 and PC-3 cell lines results may be due to the variations in the level of ALDH expressions in these cell lines, and which may be related to variable metabolic processes. Again, further validation studies are required to support this hypothesis, which could potentially pave the way for selective targeting of certain cancer types.

In both cell lines, compound **24** produced an anomalous pattern of cell viability reduction at high concentrations (50 and 100 μM in MCF7 and 50 μM in PC3), in which the combined treatment resulted in higher cell viability than the compound alone at both concentrations used. This requires further investigation to understand why this kind of combination decreased the cytotoxic effect of the compound on both cell lines. An alternate hypothesis based on the compound **24** results could be that DOX is enhancing the activity of the ALDH-affinic compounds.

Figure 5a,b represents the cytotoxic effect of DEAB on MCF7 (Figure 5a) and PC-3 (Figure 5b) cells alone and in combination with 5 μM and 1 μM DOX, respectively, compared to the single 5 μM and 1 μM DOX treatments. The results did not show a significant decrease in the cell viability for the combination treatment of DOX with DEAB in any of the DEAB concentrations used in either cell line compared to DOX alone. Although there was a reduction in cell viability of both cell lines when combination treatments were used compared to DEAB alone, the reduction was not significant compared to DOX alone. The results indicate that DEAB did not enhance the cytotoxicity of DOX on either of the tested cell lines. Taking into account the results obtained for compounds **15** and **16**, the observed effects of DEAB on both cell lines may be due to DEAB’s non-selective ALDH inhibitory activity.

Given that compounds **15** and **16** are non-cytotoxic on ALDH1A3-expressing cells even though they are potent ALDH1A3 inhibitors, the enhancement of cytotoxic activity when combined with DOX might be driven by a different mechanism. Moreover, DOX might be the agent that is enhancing the cytotoxic effects of these ALDH-affinic compounds on the tested cell lines.

The ATP-binding cassette (ABC)-superfamily is composed of several efflux pump transporters that are responsible for chemoresistance [42]. Examples of ABC transporters include P-glycoprotein (P-gp/MDR1/ABCB1), MDR-associated protein (MRP)1 (ABCC1), and breast cancer resistance protein ABCG2 (also known as BCRP, ABCP, or MXR) [43]. P-gp expression is associated with drug resistance and reduced response to chemotherapy in cancer patients, with DOX being a P-gp substrate [44,45]. Hence, the investigated compounds might be ABC substrates competing with DOX on these transporters, which would allow for enhanced DOX entry and, thus, enhanced cytotoxicity. Additionally, the lack of cytotoxicity of the potent ALDH1A3 inhibitors might be due to their ability to act as ABC transporter substrates.

Both hypotheses—ALDH-affinic compounds enhancing DOX sensitivity or vice versa, and the possibility of ALDH-affinic compounds being ABC substrates—require further investigation, along with further analog development related to compounds **15** and **16**.

## 3. Materials and Methods

### 3.1. Cell Subculture and Growth Conditions

DMEM High Glucose, RPMI-1640 media, Heat Inactivated Fetal Bovine Serum (FBS), and PBS were purchased from Euroclone, Pero, Italy. Breast cancer cell lines (MCF7 (ATCC HTB-22) and MDA-MB-231 (ATCC CRM-HTB26)) and a prostate cancer cell line (PC-3 (ATCC CRL-1435)) were purchased from ATCC^®^. MCF7 and MDA-MB-231 cells were grown in 10% (*v*/*v*) FBS/DMEM High Glucose (complete medium) and PC-3 cells were grown in 10% (*v*/*v*) FBS/RPMI-1640 (complete medium). They were incubated at 37 °C in a humidified air atmosphere of 5% CO_2_. All cells are adherent and were frequently washed with PBS and supplemented with fresh media for their growth.

### 3.2. Analysis of the Expression of ALDH1A3 in MCF7, MDA-MB-231, and PC-3 Cell Lines

Samples of MCF7, MDA-MB-231, and PC-3 cells were lysed using a RIPA lysis buffer (sc-24948A, Santa Cruz Biotechnology, Dallas, TX, USA). Equal quantities of isolated proteins were then mixed with 5× Laemmli loading dye. Proteins were separated by sodium dodecyl sulfate-polyacrylamide gel electrophoresis (SDS-PAGE) (12%) and electrophoretically transferred at a constant current of 25 V for 30 min using a Trans-Blot TurboTM Transfer System (Bio-Rad, Hercules, CA, USA) onto a polyvinylidene difluoride (PVDF) membrane, Santa Cruz Biotechnology). After blocking with 3% (*w*/*v*), fat-free milk diluted in TBST (50 mM Tris-HCl, 150 mM NaCl, pH 7.4, 0.1% (*v*/*v*) Tween 20) for 30 min at 4 °C, the membrane was incubated overnight at 4 °C with one of the following primary antibodies: anti-ALDH1A3 (ab129815; Abcam, Cambridge, UK) or anti-GAPDH antibody as a loading control (ab9485, Abcam). The amount of each antibody used was as per the manufacturer’s instructions. The next day, the primary antibody was removed, and the washing and blocking processes were carried out for 15 and 30 min, respectively. The secondary antibody (goat anti-rabbit IgG H&L (HRP) (ab205718, Abcam)) was then added for 90 min. The secondary antibody was then removed and the membrane was washed for 15 min to prepare it for imaging using a ChemiDocTM Imaging System (Bio-Rad, Hercules, CA, USA).

### 3.3. MTT Colorimetric Cell Viability Assay

Cells were seeded in 96-well plates at a seeding density of 10 × 10^3^ (MCF7), 9 × 10^3^ (MDA-MB-231), or 8 × 10^3^ (PC-3) cells/well in triplicates. Cells were incubated for 24 h to allow for cell attachment, then the media were aspirated and fresh complete media containing the compounds of interest were added. Compounds were first dissolved in DMSO (Fisher Chemicals) and then diluted in the complete medium, resulting in a final well concentration of 1% for DMSO for the highest concentration for any compound used. Untreated control samples were also prepared (1% DMSO/complete media). Final well concentrations for all compounds were 100, 50, 25, 12.5, and 6.25 μM. Plates were then incubated for 48 h. After 48 h incubation, the MTT colorimetric assay was performed as described previously [46]. Briefly, MTT was dissolved in PBS (5 mg/mL), and after 48 h, media were aspirated and discarded from all wells, and then 100 µL of fresh media was added in addition to 15 µL of MTT solution. Plates were incubated for 3 h, and then all culture media was aspirated from wells and 150 µL of absolute DMSO was added to dissolve formazan crystals. Finally, plates were incubated at room temperature while shaking for 30 min, and then absorbance was measured at 570 nm using a Multiskan GO spectrophotometer (Thermofisher, UK). Experiments were performed in triplicate, in three independent experiments. The results were then used to calculate percentage viability relative to the controls. Dose-response curves were generated using GraphPad Prism version 9.0.

### 3.4. Cell Viability Assays of Combination Treatments with Doxorubicin

Cell viability assays were conducted as described above in Section 2.3. In these experiments, DOX (Sigma-Aldrich, St. Louis, MO, USA), at a final concentration of 5 µM (MCF7 cell line) and 1 µM (PC-3 cell line) was used, along with a range of concentrations for specific compounds. Experiments were performed in triplicates, in three independent experiments.

### 3.5. Statistical Analysis

Data were presented as means and standard errors of the means (SEM). Nonlinear regression analysis was used to generate dose–response curves and determine IC_50_ values. One-way ANOVA followed by Tukey’s multiple comparisons analysis was used to compare the results of combination treatments (compounds plus DOX) with DOX alone. All statistical analyses were based on a *p* < 0.05 level of significance, using GraphPad Prism version 9.0.

## 4. Conclusions

In conclusion, several ALDH-affinic compounds were investigated as single agents and in combination with DOX on ALDH-expressing cells: breast cancer cell lines (MCF7 and MDA-MB-231) and a prostate cancer cell line (PC-3). The treatment with DOX showed enhanced cytotoxicity when combined with the selective ALDH inhibitors, particularly compound **15**, on the MCF7 cell line. Other compounds, including DEAB—a known non-selective ALDH inhibitor—did not show any promising enhancements of the DOX cytotoxicity on either cell line. Thus, the combination treatment experiment highlighted a promising potential impact of ALDH-selective inhibition in overcoming cancer resistance to chemotherapeutic agents. Indeed, the use of these ALDH-affinic compounds with DOX showed enhancement of antiproliferative activity and may be applied to overcome DOX resistance and/or to improve DOX-based therapeutic regimens.

## Figures and Tables

**Table 1:** Cytotoxicity (IC_50_ ± SEM) of **1**–**6**, **15**–**19**, **23**–**28**, **32**–**34**, and DEAB on MCF7, MDA-MB-231, and PC-3 cell lines after treatment for 48 h; n = 3.

Compound	MCF7 IC_50_ ± SEM (μM)	MDA-MB-231 IC_50_ ± SEM (μM)	PC-3 IC_50_ ± SEM (μM)
**1**	>100	>100	>100
**2**	>100	>100	>100
**3**	>100	>100	>100
**4**	>100	>100	>100
**5**	>100	>100	>100
**6**	47.0 ± 2.0	57.9 ± 2.2	50.7 ± 0.6
**15**	>100	>100	>100
**16**	>100	>100	>100
**17**	>100	>100	>100
**18**	>100	>100	>100
**19**	91.9 ± 3.4	>100	>100
**23**	>100	>100	>100
**24**	24.5 ± 0.1	31.7 ± 1.3	25.2 ± 3.6
**25**	81.4 ± 5.2	88.2 ± 1.9	62.5 ± 3.2
**26**	66.7 ± 3.4	64.8 ± 2.6	50.5 ± 2.7
**27**	83.5 ± 0.4	>100	>100
**28**	63.4 ± 0.5	63.9 ± 2.5	48.7 ± 0.5
**32**	65.4 ± 5.6	63.1 ± 4.9	48.8 ± 1.2
**33**	>100	81.9 ± 12.2	67.6 ± 1.5
**34**	>100	>100	>100
DEAB *	>100	>100	>100

* DEAB: diethylaminobenzaldehyde.

## Data Availability

Data are contained within the article.
